# Respiratory syncytial virus in severe lower respiratory infections in previously healthy young Ethiopian infants

**DOI:** 10.1186/s12887-021-02675-3

**Published:** 2021-04-28

**Authors:** Abate Yeshidinber Weldetsadik, Frank Riedel

**Affiliations:** 1 Pediatric Pulmonologist, St. Paul’s Hospital Millennium Medical College, Addis Ababa, Ethiopia; 2 Pediatric Pulmonology, University Medical Center Hamburg-Eppendorf, Hamburg, Germany

## Abstract

**Background:**

Respiratory Syncytial Virus (RSV) is the commonest cause of acute lower respiratory infections (ALRI) in infants. However, the burden of RSV is unknown in Ethiopia. We aimed to determine the prevalence, seasonality and predictors of RSV infection in young infants with ALRI for the first time in Ethiopia.

**Methods:**

We performed RSV immuno-chromatographic assay from nasopharyngeal swabs of infants, 29 days to 6 months of age. We included the first 10 eligible infants in each month from June 2018 to May 2019 admitted in a tertiary pediatric center. Clinical, laboratory and imaging data were also collected, and chi-square test and regression were used to assess associated factors with RSV infection.

**Results:**

Among a total of 117 study children, 65% were male and mean age was 3 months. Bronchiolitis was the commonest diagnosis (49%). RSV was isolated from 26 subjects (22.2%) of all ALRI, 37% of bronchiolitis and 11% of pneumonia patients. Although RSV infection occurred year round, highest rate extended from June to November. No clinical or laboratory parameter predicted RSV infection and only rainy season (Adjusted Odds Ratio (AOR) 10.46 [95%. C.I. 1.95, 56.18]) was independent predictor of RSV infection.

**Conclusions:**

RSV was isolated in a fifth of young infants with severe ALRI, mostly in the rainy season. Diagnosis of RSV infection in our setting require specific tests as no clinical parameter predicted RSV infection. Since RSV caused less than a quarter of ALRI in our setting, the other causes should be looked for in future studies.

## Introduction

Acute lower respiratory infections (ALRI) are common causes of morbidity and mortality in under-five children with the highest burden in sub-Saharan Africa [1]. Respiratory Syncytial Virus (RSV), Pneumococcus, Haemophilus influenza, and Influenza virus are among the most common etiologies [1]. RSV was responsible for 31% of all ALRI in the recent Pneumonia Etiology Research for Child Health (PERCH) study from low and middle income countries [2]. RSV and other respiratory viruses are playing an increasing role in ALRI as bacterial infections are effectively controlled by vaccines [2, 3]. RSV is generally associated with up to 28% of all ALRI and 13–22% of all mortality in young children [4].

Although RSV can affect all ages, almost all children are infected by 2 years of age [5]. RSV related hospitalization and case fatality rate is, however, highest in infants younger than 6 months with 45% of admissions and deaths occurring in this age group [4, 6].

RSV is associated with variety of clinical conditions from asymptomatic and mild upper respiratory infection to life threatening pneumonia and bronchiolitis [7]. It is also associated with increased risk of asthma and impaired lung function later on [8]. Risks for severe infection include prematurity, low birth weight, male sex, presence of siblings, maternal smoking, history of atopy, lack of breastfeeding, crowding and comorbidities, especially chronic neonatal lung disease and congenital heart defects [4, 9–12].

Despite different case definitions for detection of confirmed RSV-ALRI, specificity and sensitivity varies widely with consistently low positive predictive value [13, 14]. Thus, diagnosis of RSV infection usually requires the use of microbiologic tests [14]. However, if we could clinically predict the probability of viral ALRI precisely, it would be of importance for rational use of antibiotics, a common problem in our settings [15].

RSV and other viral epidemiologic studies are important to determine disease burden, need of diagnostic and future vaccination services, and assess risk factors to protect high risk populations [8, 16, 17]. It also helps to design preventive and therapeutic interventions for households, hospitals and health planners by determining RSV seasonal variation in different regions [18, 19]. The epidemiology and seasonality of RSV have been the focus of many investigations. RSV prevalence rates from previous studies in infant ALRI ranged from 9% in Nepal to more than 60% in Ghana although the different population of children and methods makes comparison difficult [10, 18, 20]. Other African studies reported also low prevalence rates in Kenya (12%), Senegal (11.4%), Zambia (15.2%), and Burkina Faso (12%) [1, 20–22].

Although RSV is one of the most extensively studied causes of ALRI in humans, most studies come from high income countries (46% from North America) [23]. Research in low and middle income countries are however both limited and none from Ethiopia [23–25]. Thus, we essentially aimed to determine the prevalence and season of RSV infection and factors associated with RSV in infants less than 6 months of age with severe ALRI for the first time in Ethiopia.

## Materials and methods

### Study setting

The study was done in the Pediatric Department of Saint Paul’s Hospital Millennium Medical College (SPHMMC) in Addis Ababa, capital city of Ethiopia. The hospital is a tertiary academic and referral center for a population of 8 million. The Department of Pediatrics has currently 114 beds (48 Neonatal beds, 60 pediatric, and 6 pediatric ICU beds) and provide care to children from birth to 15 years of age.

### Sampling method

Study subjects were selected at admission with ALRI to SPHMMC after checking eligibility criteria and taking written informed consent, starting from June 2018 to May 2019. In each month of the year, the first 10 consecutive eligible cases were tested for RSV antigen.

### Data collection and procedures

A nasopharyngeal swab was collected from each infant at the bedside by a trained nurse within 48 h of hospital admission, using a flexible sterile applicator. The specimen was then immediately tested for presence or absence of RSV fusion protein using RSV immuno-chromatographic assay (Alere™, USA, Scarborough).

This assay is a standardized point of care test which is read after 15 min and gives the result as “RSV positive”, “RSV negative” or “Invalid”. The test detects RSV fusion protein from both RSV A and B subgroups. Sensitivity and specificity of the test was reported to be 93% compared to gold standard viral culture [26].

We tested 120 infants, 29 days to 6 months of age, with ALRI for RSV antigen with this test, no invalid result was found during the study.

Socio-demographic, clinical, laboratory and imaging data of all patients were documented using a semi–structured questionnaire, data were collected by two research nurses working in pediatric emergency and wards after training on data collection and the RSV testing procedure with close monitoring by the principal investigator.

Data analysis was done using SPSS for Windows Version 20 (SPSS Inc., Chicago, IL) and both descriptive and analytical methods were used as needed, a *p*-value of less than 0.05 was taken as significant. Multivariate analysis was applied to control confounders and variables with *p* value of < 0.2 on bivariate analysis, and previously known predictors of RSV infection were included in multivariate logistic regression.

### Inclusion criteria

- Infants 29 days - 6 months of age
- Upper respiratory symptoms in the preceding 7 days
- Clinical sign of severe ALRI requiring hospitalization

### Exclusion criteria

- Underlying chronic illness (Cardiac, neurologic, and lung diseases, prematurity and immunodeficiency including HIV infection or exposure)
- Infants with Severe Acute Malnutrition (defined by weight for length < − 3 SD or nutritional edema)

### Operational definition

- Severe ALRI: Acute onset (less than 7 days) with respiratory symptoms (cough, dyspnea, wheezing, tachypnea, grunting…) requiring hospitalization
- Bronchiolitis: ALRI with first wheezing with or without bilateral fine crackles in an infant with preceding upper respiratory infection with or without hyperinflation and atelectasis in chest x-ray.
- Pneumonia: WHO definition excluding those fulfilling the diagnosis of bronchiolitis
- Bronchitis: ALRI with cough, mild / no tachypnea, rhonchi or coarse crackles on auscultation with or wthout peribronchial cuffing on chest x-ray in an infant with preceding viral upper respiratory infection
- Overcrowding: Presence of more than two persons per room of total rooms
- Young infants: 29 days to 6 months of age.

## Results

### Patient characteristics

A total of 117 infants were included in the final analysis out of 120 infants tested for RSV (three were excluded later after a delayed diagnosis of underlying comorbidity: one primary immunodeficiency, one cardiac malformations and one chromosomal abnormality).

Male sex predominated (65%) with male/female ratio of 1.8, and Mean age was 3 (SD ± 1.8) months. Most mothers (56%) were uneducated or completed only primary education. Overcrowding was found in 58% households, cigarette smoking was uncommon (2.6% of households), exclusive breast feeding was documented in 54% of cases (Table 1).

**Table 1:** Sociodemographic and clinical data (*n* = 117)

Sociodemographic data: n (%)			Clinical Presentation: n (%)		
Age	1–3 mo.	63 (53.8)	Respiratory distress	Yes	113 (96.6)
	4–6 mo.	54 (46.1		No	3 (2.6)
Sex	male	76 (65)		n.k.	1 (0.9)
	female	41 (35)	Temperature	Normal	50 (42.7)
Family size (No)	< 4	59 (50.4)		Low fever	49 (41.9)
	5–7	47 (40.2)		High fever	14 (12)
	> 7	11 (9.4)		n.k.	4 (3.4)
Maternal education	No/Primary	66 (56.4)	Auscultation	Clear chest	13 (11.1)
	High school	21 (17.9)		Wheeze or crackles	103 (88)
	College	9 (7.7)		n.k.	1 (0.9)
	n.k.	21 (17.9)	Bulging chest	Yes	7 (6)
Residency	Urban	90 (76.9)		No	107 (91.5)
	Rural	22 (18.8)		n.k.	3 (2.6)
	n.k.	5 (4.3)	Dehydration	Yes	7 (6)
Overcrowding	Yes	63 (58.3)		No	92 (78.6)
	No	48 (41)		n.k.	18 (15.4)
	n.k.	6 (5.1)	PICU admission	Yes	4 (3.4)
Household smoking	Yes	3 (2.6)		No	112 (95.7)
	No	98 (83.8)	WBC (/μl)	< 5.000	2(1.7)
	n.k.	16 (13.7)		5.000–15.000	94 (80.3)
Infant feeding	EBF	63 (53.8)		15.000–20.000	11 (9.4)
	Bottle	13 (11.1)		> 20.000	8 (6.8)
	Mixed	25 (21.4)	CRP	Low	57 (48.7)
	n.k.	16 (13.7)		Positive / high	26 (22.2)
Sun exposure	Yes	68 (58.1)		n.k.	34 (29.1)
			CXR	Normal	19 (16.2)
	No	48 (41)		Pneumonia	6 (5.1)
				Bronchiolitis	21 (17.8)
	n.k.	1 (0.9)		Not done	59 (50.4)
Monthly income (in ETB)	< 2000		Blood culture	n.k. or not done	114 (97.4)
	> 2000				
	n.k.			positive culture	3 (2.6)

Keys: *n* number of cases, *n.k* not known, *EBF* exclusive breast feeding, *PICU* pediatric intensive care unit, *WBC* white blood count, *CRP* C reactive protein, *CXR* chest x-ray, *ETB* Ethiopian Birr

### Clinical presentation

Low fever (axillary temperature 37.5–38.3 °C) and no fever (36–37.4 °C) were documented in 42% each. Nearly all (97%) were in respiratory distress. Crackles or wheeze were found in 83% of infants on auscultation. Pediatric ICU admission was required in 3.4% of the cases for respiratory failure (Table 1).

### Laboratory and imaging characteristics

WBC was done in all infants and was normal in 80% of cases (Table 1). Most (71%) had CRP documented and elevated in 22% and was normal or low in 49% of infants. Almost 30% of all CRP > 60 mg/L were documented in RSV confirmed cases (Table 1). Blood culture was positive in 3 infants (2.6%) (first, a 4 months RSV positive infant with bronchiolitis had hospital acquired infection, second, a 2-month infant with RSV negative pneumonia and hospital acquired sepsis and DIC, isolated multidrug resistant Acinetobacter and *K. pneumoniae*, ventilated in the PICU, had mild pleural effusion on chest Ultra Sound, and died of severe sepsis, the third is 4-month infant with unspecified organism). Only half of the infants had chest x-ray done and 33% were normal while 36% showed hyperinflation and/ peribronchial thickening or atelectasis. Only 10% of the x-rays showed isolated consolidation or infiltrate consistent with pneumonia (Table 1).

### Type of ALRI and RSV infection

**Fig. 1:**
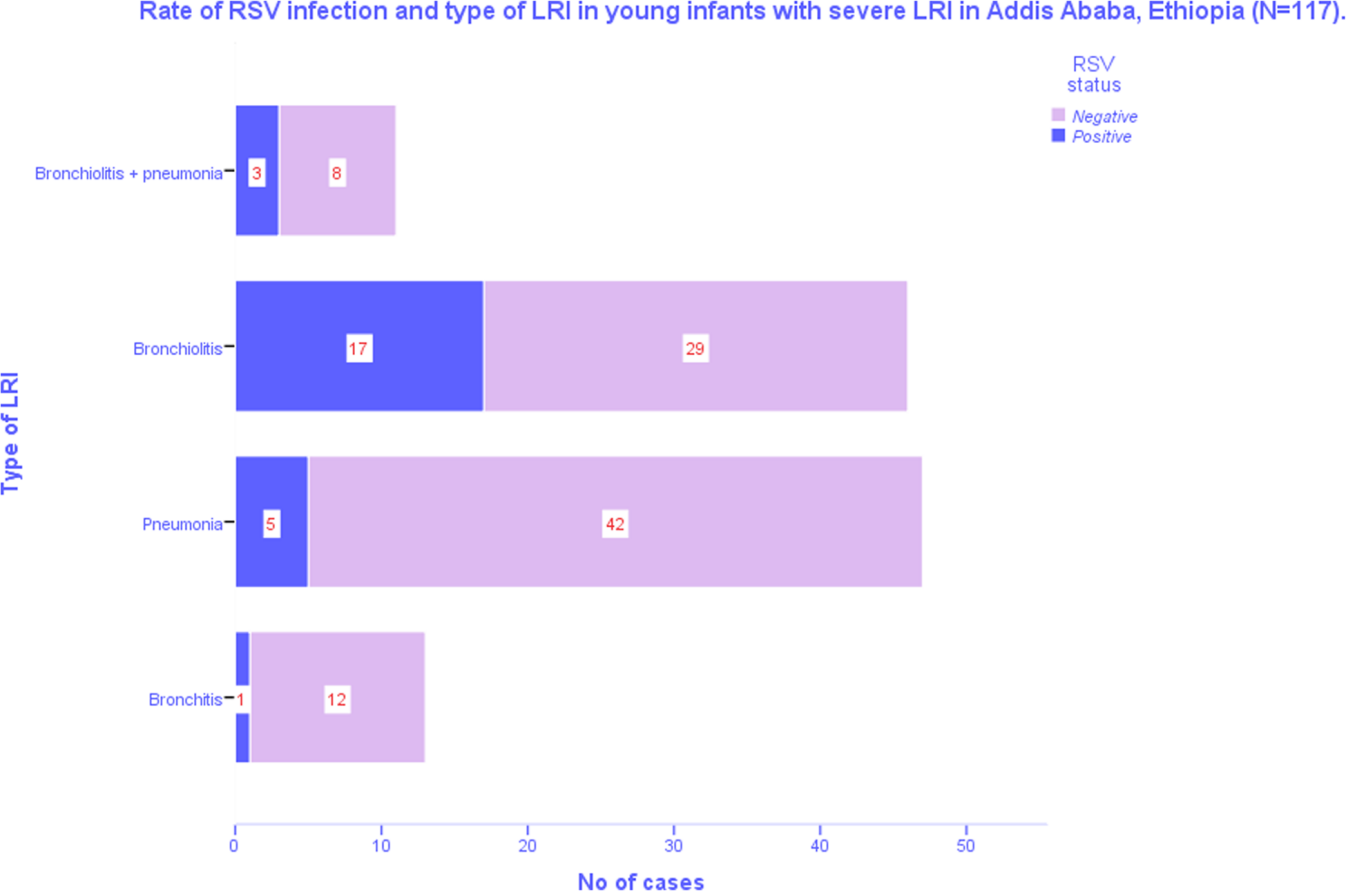
Rate of RSV infection and type of Lower Respiratory Infections (LRI), (*n* = 117)

**Fig. 2:**
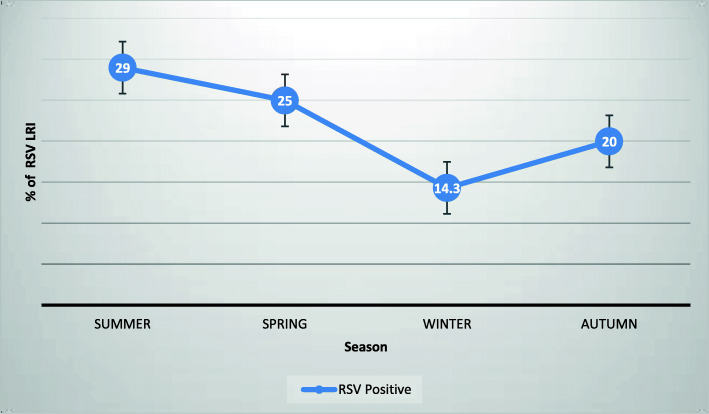
Seasonal variation of RSV in % of RSV in Lower Respiratory Infections (LRI)

Bronchiolitis (48.7%) followed by pneumonia (40%) were the commonest ALRI (Fig. 1, Table 2). RSV infection was documented in 26 (male/female ratio of 1) of all young infants less than 6 months (22.2%), but in 37% of those with bronchiolitis and only 11% of those with pneumonia. Females (31.7%) were slightly more affected by RSV than males (17.1%), but this was not statistically significant (*p* = 0.11). Most (58%) of the RSV infections were documented during rainy season. Although RSV infection was documented year round, the highest rate of infection occurred in the local summer time (June–August) followed by spring (September to November), while the lowest was in winter (December to February) (Fig. 2).

**Table 2:** Diagnosis, treatment and outcome (*n =* 117)

Diagnosis and treatment		n	%	Outcome		n	%
Type of LRI	Bronchiolitis	57	48.7	RSV	Positive	26	22.2
					Negative	91	77.8
				LOS (*n =* 88)	≤ 3 days	34	38.6
					4–7 days	33	36.4
	Pneumonia	47	40.2		7–14 days	21	25
	Bronchitis	13	11.1	Outcome	Complete resolution	115	98.3
Supportive therapy	Oxygen	117	100				
	Hydration	117	100				
	Bronchodilator	42	36				
	Epinephrine nebulization	33	28				
					Persistent atelectasis	1	0.8
Steroids	Yes	12	10				
	No	103	88		PPE / sup- puration	1	0.8
Antibiotics	No antibiotics	29	24.8		Air leak	0	0
	Ampicillin	26	22.2				
	Ampicillin + Gentamicin	30	25.6		Death	1	0.9
	Cephalosporin	25	21.4				
	Vancomycin ± Cephalosporin	6	5				

Key: *LOS* length of hospital stay in days, *PPE* parapneumonic effusion

### Treatment and outcome

The mean length of hospital stay was 5 (SD ± 5.2) days, ranging from 1 to 33 days. Most infants (75%) were treated with antibiotics, while 25% received only supportive care with hydration and oxygen. Bronchodilator and epinephrine nebulization were used in 48% infants. Steroids (mainly dexamethasone) were given in 10% of cases. Ampicillin +/− gentamicin was used in half of the cases and a quarter of the infants received broader spectrum antibiotics, mainly Cephalosporines, and vancomycin was also used in 5% of the cases.

Resolution of ALRI was documented in 98.3% with only 2 cases having complications, both of which were RSV negative: A one-month old infant with bronchiolitis developed persistent atelectasis and required a repeat admission for the same complaint in 4 weeks, and one death (0.9%) happened, with a diagnosis of pneumonia and sepsis, the latter being the cause of death (Table 2).

### Epidemiology and predictors of RSV infection

**Fig. 3:**
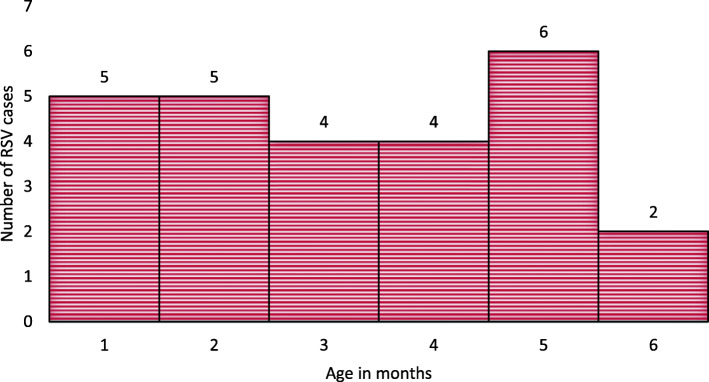
RSV positive cases distributed by age group (*N* = 26)

ALRI season, presence of wheeze, and bulging chest, and the clinical diagnosis of bronchiolitis were significantly associated with RSV isolation on bivariate analysis. However, on multivariate regression, only the season was found to be independent predictor of RSV infection with adjusted Odds Ratio (AOR) of 9.15 (95% C.I. 1.30, 64.32) (Fig. 2, Table 3). Other variables including sociodemographic, clinical, laboratory, and imaging parameters and outcome were not associated with RSV infection (Fig. 3, Tables 3 and 4) in this group of young infants less than 6 months of age.

**Table 3:** Bivariate and multivariate analysis of factors associated with RSV infection (*n* = 117)

	Variable	p value	COR (95% C.I)	p value	AOR (95% CI)
1	Age	0.11	–	0.06	13.5 (0.89,206)
2	Sex	0.07	2.25 (0.93,5.47)	0.06	0.29 (0.08,1.06)
3	Presence of wheeze	0.001	5.10 (1.97,13.02)	0.17	3.33 (0.53,20.97)
4	Type of LRI	0.002	4.87 (1.78,13.27)	0.69	0.53 (0.09,3.22)
5	Season of LRI	0.02	2.92 (1.20,7.13)	0.02	9.15 (1.30,64.32)
6	Bulging chest	0.04	5.33 (1.11,25.56)	0.25	4.62 (0.33,63.93)
7	Infant feeding	0.12	–	0.51	4 (0.54,29.7)
8	WBC	0.34	–	0.99	–
9	CRP	0.52	0.66 (0.23,1.91)	0.47	0.50 (0.08,3.12)
10	CXR	0.78	–	0.48	1.85 (0.33,10.38)

Key: *COR* crude odds ratio, *AOR* adjusted odds ratio, *CI* confidence interval

**Table 4:** Clinical characteristics of RSV positive young infants with LRI (*n* = 26)

Variables	n	Percent
Age in months		
1	5	19.2
2	5	19.2
3	4	15.4
4	4	15.4
5	6	23.1
6	2	7.7
Sex		
male	13	50
female	13	50
Feeding		
EBF	17	65.4
Mixed	4	15.4
Wheeze		
Yes	18	69.2
No	8	30.8
WBC		
Normal	24	92.3
15.000–20.000 /μl	2	7.3
CRP		
< 36 mg/L	15	57.7
36-60 mg/L	2	7.7
> 60 mg/L	4	15.4
Season		
Summer	9	34.6
Spring	7	26.9
Winter	4	15.4
Autumn	6	23.1
Dx		
Bronchitis	1	3.8
Pneumonia	5	19.2
Bronchiolitis	20	77

Key: *WBC* white blood count, *CRP* C reactive protein, *Dx* Diagnosis

## Discussion

The epidemiology of viral respiratory infections is not well documented in Ethiopia, except influenza virus surveillance since 2008 [27, 28], and to our knowledge, this is the first study evaluating the burden of RSV infection among young infants less than 6 months of age with ALRI in the country. Similar to previous studies [12, 13], the clinical presentation was not different between RSV positive and negative infants except for higher rate of wheezing in those with RSV infection. The initial presentation was dominated by respiratory distress, while high fever was uncommon in our patients. The absence of a special clinical predictor for RSV infection in our setting indicates the need to use virus specific tests if we want to diagnose RSV infection with certainty as also shown in the PERCH study [12, 14].

Our data showed that RSV plays an important role as a cause of severe ALRI in Ethiopian infants. We also demonstrated that RSV is much more prevalent than influenza in young children with ALRI, for whom a 3% rate was documented previously in Addis Ababa [27].

In contrary to the high RSV rate in western settings [29] and a study from Ghana [18], we documented RSV infection in only a fifth of all infant ALRI and about a third of bronchiolitis cases.RSV prevalence in our study was similar to a study in Maputo [30], but higher than other studies documenting 9–15% prevalence [10, 20–22]. While the strict exclusion of risk populations may affect our prevalence rate, the younger age of our patients (less than 6 months) may also be the reason for higher prevalence compared to the previous African studies [20–22]. Additionally, other viruses and bacteria probably play a more important role in developing countries compared to western settings, where RSV has a much higher importance than other viruses and bacteria [2, 29, 31]. However, vaccination was up to date and almost all infants had a preceding upper respiratory infection in our study, thus bacterial etiologies were unlikely in the majority of the patients, although it was not explored adequately.

Male gender was found to be a risk for ALRI and our result of male/female ratio of 1.8 is in line with this observation [32]. However, male gender preference and more health care seeking in males had also been recognized in other developing countries [33]. There was no gender difference in the RSV rate in our study, although male gender has been documented as a risk factor in previous studies [34].

Only the rainy season of the year was independently associated with RSV infection in our study. Although RSV infection was documented year round, the highest rate was found from June to November which is the rainy season in Addis Ababa. Although we didn’t measure meteorological factors in our study, this time is associated with cold weather and high air pollution (PM 2.5) in Addis Ababa and coincides with higher rates of respiratory infection and admissions in young children [35]***.*** Recent studies have documented the role of air pollution in RSV infection [36]. RSV seasonality had also been typically associated with rainy season in tropical regions, but dry season in temperate zone [19, 37]. While the association of the RSV peak with rainy season is explainable by our tropical climate, air pollution and cold weather during this time, we however cannot determine seasonality as our study covered a period of single year.

Diagnosis of viral ALRI can prevent unnecessary antibiotic treatment, especially in previously healthy children with low risk of bacterial infection. Serious bacterial infections are rare in those previously healthy and vaccinated children with bronchiolitis [38]. One-fourth of our patients were not treated with antibiotics, but there was no difference in RSV positive compared to RSV negative infants in use of antibiotics. The outcome of infants treated with antibiotic versus supportive care alone was also similar. Our study also showed that judicious withhold of antibiotics can be practiced in severe ALRI where a viral etiology is most likely, without compromising outcome - at least in specialized centers of resource limited settings, especially in children with updated vaccinations and without comorbidity [39].

Antibiotic choice was not strictly followed as per standard recommendations, with a third of patients receiving highly broad spectrum antibiotics (mostly third generation Cephalosporines). Despite their irregular availability in low income settings and their inherent limitations, point of care tests should contribute in the future to decrease antibiotic use, indicate the choice of antibiotic and avoid both over and under treatment of children with ALRI [12]. Antibiotics are not indicated as prophylactic management in viral ALRI, and RSV infection has been shown to be rarely complicated by bacterial supper infection [12, 38, 40].

Complications were low in both RSV positive and RSV negative infants in our study. This could be due to the predominantly viral etiology of the ALRI in our patients as evidenced by preceding cold, low fever, mostly normal WBC and CRP. Furthermore, most infants had bronchiolitis and only 10% of CXRs confirmed pneumonia. The low mortality (0.9%) is also explained by exclusion of infants with comorbidities and with the rarity of death in previously healthy infants with ALRI receiving proper treatment [40].

Our study has some limitations. First, we used antigen detection without a molecular or culture confirmation. Additionally, our low sample size and inclusion of selected severely ill cases from a single tertiary center limits generalizability and identification of predictors of RSV infection in our population of young infants. RSV prevalence in the general pediatric population in our setting is also not possible from our study and this will require a separate study with inclusion of older children and milder cases. We also could not determine seasonality exactly because of a single year coverage. Another limitation of our study design is that it cannot doubtlessly prove a direct causation relation of RSV being the cause of the ALRI, although previous case control studies had documented that RSV isolation from upper airways reflect genuine ALRI caused by RSV, unlike by other viruses and bacteria [2, 19].

These limitations of ours may be addressed in future studies by conducting multicenter case control studies using multiplex-PCR for more comprehensive description of ALRI etiologies in Ethiopia.

In conclusion, our study showed that RSV is a common cause of ALRI in young Ethiopian infants less than 6 months of age. The rainy season is associated with the highest RSV infection unlike in temperate zones. No clinical, laboratory or imaging parameters predict RSV infection in our setting in a highly selected young infants less than 6 months of age, underscoring the paramount need for RSV specific tests to confirm the diagnosis in order to reduce the abuse of antibiotics among these young infants presenting with ALRI.

## Data Availability

Data can be provided from the PI (AYW) with a request.

## Abbreviations

ALRI: Acute Lower Respiratory Infection
CI: Confidence Interval
CRP: C-Reactive Protein
OR: Odds Ratio
AOR: Adjusted Odds Ratio
HIV: Human Immune deficiency Virus
ICU: Intensive Care Unit
LRI: Lower Respiratory Infection
PCR: Polymerase Chain Reaction
RSV: Respiratory Syncytial Virus
SPHMMC: Saint Paul’s Hospital Millennium Medical College
WBC: White Blood Cell count
WHO: World Health Organization
